# Peculiar contact dermatitis in a construction worker

**DOI:** 10.1111/cod.13145

**Published:** 2018-10-29

**Authors:** Lieke C. J. van Delft, Nadine A. M. Ramakers, Valerie L. R. M. Verstraeten

**Affiliations:** ^1^ Department of Dermatology Maastricht University Medical Centre+ Maastricht The Netherlands; ^2^ Dermadok Huidkliniek Antwerp Belgium

**Keywords:** case report, chromium, construction industry, contact dermatitis, manganese, nickel

Construction workers often have their lower backs exposed because of a crouched working position and release of the shirt. This makes them vulnerable to occupational contact dermatitis caused by metal, leather and other materials present in a working material holster belt directly rubbing on the lower back skin. The itching and inevitable scratching may lead to work absenteeism and loss of productivity.^1^, ^2^

## CASE REPORT

A 43‐year‐old scaffolder consulted with a 5‐year history of itchy red skin on the lower back. During working hours, he wore a leather belt with a metallic hammer, level gauge, and ratchet. He also wore a non‐leather girdle strapped between the legs, around the waist and over the shoulders with which he was attached to the building; these areas of the skin were not affected. He wore textile working gloves with rubber grips on the outer surfaces of the gloves at the palms most of the time, working shoes, trousers, and a tucked‐in shirt; the latter tended to roll up while he was working. He had no medical history, was not taking medication, and did not have an atopic constitution.

**Figure 1:**
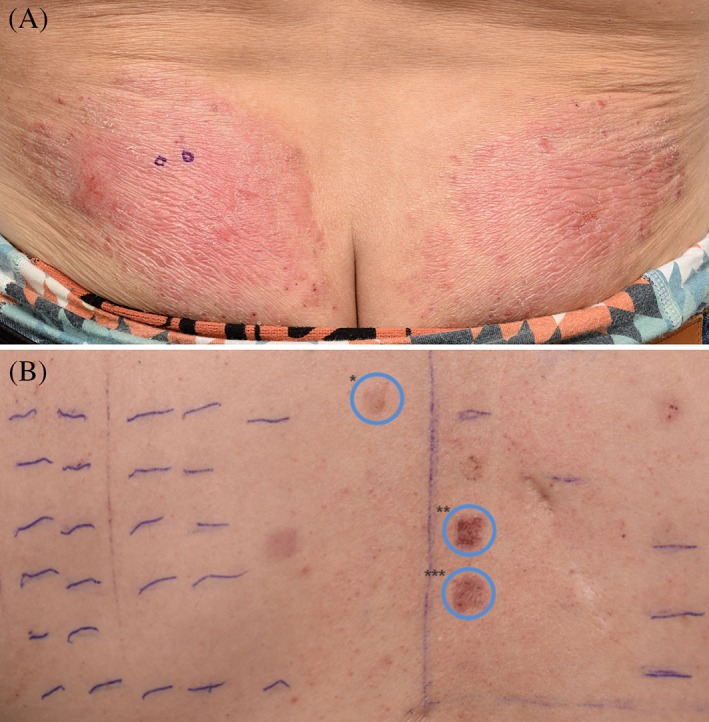
(**A**) Palm‐sized erythematous lichenified, partly excoriated plaques symmetrically located on the lower back and nates. (**B**) Indurated erythematous plaques on the upper back indicating a positive patch test results for: *manganese dichloride 2%, **potassium dichromate 0.5% and ***nickel(II) sulfate hexahydrate 2% all in petrolatum

We observed palm‐sized erythematous lichenified, partly excoriated plaques symmetrically located on the lower back and nates, and erythematous hyperkeratotic patches on the entire surfaces of both palms and soles (Figure 1A). Histological evaluation of punch biopsy material from the plaque on the nates showed spongiotic dermatitis; culture of the biopsy specimen showed no growth of yeast, fungi, or atypical mycobacteria. Treatment was initiated with betamethasone 0.05% and salicylic acid 3% ointment for the palms and soles, and clobetasol 0.05% ointment for the lower back and nates. After 2 months, the skin lesions significantly improved with this therapy and sick‐leave. Patch tests were then conducted with the TRUE Test and our baseline extension, a shoe and a metal series, in Van der Bend square chambers (Brielle, the Netherlands) attached with Medipore (Maplewood, Minnesota) tape. Patches were removed after 2 days; the skin reaction was evaluated on day (D) 4 in accordance with the ICDRG/ESCD guidelines.^3^ The initial patch test was completely negative, but heavy perspiration was visible on the tested site. A false‐negative result was suspected, and the test was repeated a few weeks later. We found clearly positive patch test results in the metal series for nickel sulfate 2% pet. (D2, ++; D4, ++), manganese dichloride 2% pet. (D2, +; D4, +), and potassium dichromate 0.5% pet. (D2, ++; D4, ++) (Figure 1B).

This led us to assume that the patient had occupational contact dermatitis caused by potassium dichromate in the leather tool‐belt and by nickel that was probably present in the tools. Regrettably, because of long‐term sick‐leave, the patient could not supply the tools to us for verification of the presence of nickel. Since 2014, the maximum amount of chromium(VI) allowed in leather articles that come into contact with skin has been restricted to 3 mg/kg by order of the EU.^4^ For assessment of the level of chromium in the tool‐belt, the proper test would be the diphenylcarbazide test; however, this requires high‐performance liquid chromatography equipment, which we do not possess. Chromium spot tests do not seem to be as reliable. It is thus not clear whether the belt released >3 mg/kg of chromium(VI). Because of therapy resistance and remission upon sick‐leave, we strongly suspected chromium and nickel to be clinically relevant contact allergens in this case.

Thus, the patient was advised, when resuming work, to use non‐leather working gloves at all times, change the belt to a non‐leather alternative, and ensure that the lower back remained unexposed to the belt and tools by wearing a longer shirt and/or by lining the inside of the leather tool‐belt with cotton.

## DISCUSSION

In conclusion, our clinical suspicion of occupational contact dermatitis was high, although the first round of patch tests gave negative results. We suspected a false‐negative result attributable to excessive perspiration, and performed the patch tests again, with positive results. When skin reactions on the lower back are observed in construction workers, one should bear in mind the crouched working position, and exposure of the skin on the lower back to metal and leather materials. The prevalence of contact dermatitis among construction workers is high, and patients may not relate work materials and tools to their skin problems.^5^

## CONFLICT OF INTEREST

The authors declare no potential conflict of interests.

## Supporting information


**Figure S1** Indurated erythematous plaques on the upper back indicating positive patch test results for: *manganese dichloride 2% pet., **potassium dichromate 0.5% pet., and ***nickel sulfate 2% pet.
