# Supplementary material for: Peculiar contact dermatitis in a construction worker
Source: Contact Dermatitis. 2018 Oct 29;80(3):166–7. doi: 10.1111/cod.13145 (PMC6587838; doi:10.1111/cod.13145)
Supplement: Supplementary file 1 — Figure S1 Indurated erythematous plaques on the upper back indicating positive patch test results for: *manganese dichloride 2% pet., **potassium dichromate 0.5% pet., and ***nickel sulfate 2% pet. [file COD-80-166-s001.docx]

| ­­­­­ 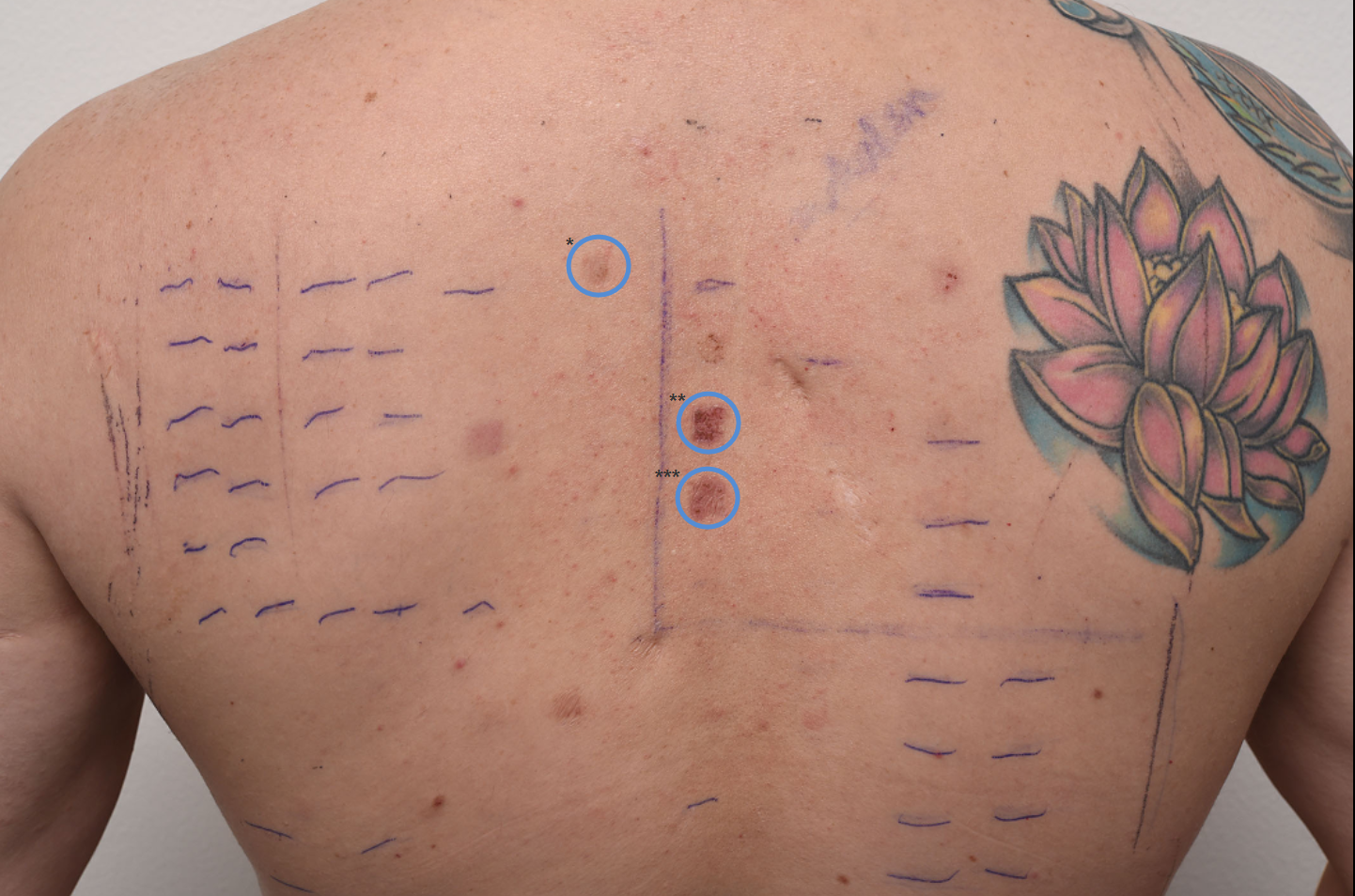  **Figure S1:** Indurated erythematous plaques on the upper back indicating a positive patch test results for: *manganese dichloride 2% pet., **potassium dichromate 0.5% pet. and ***nickel sulfate 2% pet.. |
| --- |
